# Biomarker for renal scarring screening in children with vesicoureteral reflux: a systematic review

**DOI:** 10.3389/fped.2025.1621716

**Published:** 2025-08-26

**Authors:** Utari Mudhia Arisa Putri, Putu Angga Risky Raharja, Gerhard Reinaldi Situmorang, Irfan Wahyudi, Arry Rodjani, Henny Adriani Puspitasari, Abubakr Imam, Luis R. Saraiva, Santiago Vallasciani, Tariq O. Abbas

**Affiliations:** ^1^ Urology Department, Cipto Mangunkusumo Hospital/Faculty of Medicine, Universitas Indonesia, Jakarta, Indonesia; ^2^ Pediatric Nephrology Division, Sidra Medicine, Doha, Qatar; ^3^ Translational Medicine Division, Research Branch, Sidra Medicine, Doha, Qatar; ^4^ Department of Comparative Medicine, Yale University School of Medicine, New Haven, CT, United States; ^5^ College of Health & Life Sciences, Hamad Bin Khalifa University, Doha, Qatar; ^6^ Urology Division, Sidra Medicine, Doha, Qatar; ^7^ Weil Cornell Medicine–Qatar, Cornell University, Doha, Qatar; ^8^ College of Medicine, Qatar University, Doha, Qatar

**Keywords:** biomarkers, NGAL, renal scarring, vesicoureteral reflux, systematic review

## Abstract

**Introduction:**

Vesicoureteral reflux (VUR) is a prevalent pediatric urological condition that increases children's risk of urinary tract infections (UTIs) and renal damage. Renal scarring linked to VUR can lead to long-term complications, including hypertension and chronic kidney disease (CKD). Although traditional imaging techniques, such as dimercaptosuccinic acid (DMSA) scans, are regarded as the gold standard for identifying renal scarring, they come with risks of radiation exposure and high costs. This review investigates the diagnostic accuracy of blood and urine biomarkers as alternative methods for detecting renal scarring in VUR.

**Methods:**

This systematic review adhered to the PRISMA 2020 guidelines. We conducted a comprehensive search across three databases—PubMed, ScienceDirect, and Cochrane—for studies on biomarkers associated with renal scarring in children with VUR. The included studies were evaluated for diagnostic accuracy (sensitivity and specificity) and assessed for risk of bias using the QUADAS-2 framework.

**Results:**

Nine studies met the eligibility criteria and were included in the qualitative synthesis. Biomarkers such as NGAL, CRP, CXCL8/IL-8, LL-37, and IL-6 were evaluated. Among these, urinary NGAL demonstrated the best diagnostic performance, with sensitivity ranging from 72%–84% and specificity between 60% and 81%. Other biomarkers exhibited moderate accuracy, although they were less reliable than NGAL. Overall, biomarkers present a promising non-invasive alternative to traditional imaging for detecting renal scarring in children with VUR.

**Conclusion:**

Urinary biomarkers, particularly NGAL, hold potential for detecting VUR and renal scarring in children, providing a non-invasive alternative to traditional imaging methods. However, additional validation and standardization are necessary before these biomarkers can be routinely applied in clinical practice.

## Introduction

Vesicoureteral reflux (VUR) is a common urological condition in children characterized by the retrograde flow of urine from the bladder to the ureters or kidneys, which predisposes them to recurrent urinary tract infections (UTIs) and potential renal parenchymal damage and scarring. Among children diagnosed with VUR, the risk of renal scarring is a critical concern, as it can lead to long-term complications such as hypertension and chronic kidney disease (CKD) (1, 2). Early identification and monitoring of renal scarring are essential to mitigate these risks and guide appropriate clinical interventions.

Conventional imaging techniques, such as the dimercaptosuccinic acid (DMSA) scan, are regarded as the gold standard for detecting renal scarring. However, DMSA is not sufficiently accurate to detect scars of all grades (3). Additionally, DMSA scanning has notable limitations, including radiation exposure, limited availability in certain clinical settings, and high costs, which make it less suitable for routine screening (4). In response to these challenges, there is growing interest in using non-invasive biomarkers derived from blood or urine as a promising alternative for screening and diagnosing renal scarring in children with VUR.

Biomarkers found in urine or blood, such as neutrophil gelatinase-associated lipocalin (NGAL), kidney injury molecule-1 (KIM-1), and inflammatory cytokines, provide potential advantages, including minimal invasiveness, ease of collection, and the ability to reflect real- time pathophysiological changes (5). Studies evaluating these biomarkers have reported varying degrees of accuracy in detecting renal scarring, with some demonstrating high sensitivity and specificity. However, the absence of consensus regarding the most effective biomarkers, along with variability in study methodologies and populations, underscores the need for a systematic review to synthesize current evidence and assess their diagnostic utility. This systematic study aims to evaluate the precision of blood and urine biomarkers in screening for renal scarring in children with VUR. By analyzing and summarizing data from existing studies, this review aims to provide a comprehensive understanding of the diagnostic potential of biomarkers and inform future clinical practices.

## Methods

This systematic review was conducted following the 2020 Preferred Reporting Items for Systematic Reviews and Meta-Analyses (PRISMA) guidelines to ensure transparency and reproducibility (6). This systematic review focuses on children with vesicoureteral reflux (VUR), a condition that increases the risk of renal scarring. It evaluates the diagnostic performance of urinary and blood biomarkers—such as NGAL, CRP, IL-6, and LL-37—as potential non-invasive alternatives to traditional imaging techniques like DMSA scans. The primary outcome assessed is the accuracy of these biomarkers, using sensitivity, specificity, and AUC values, to determine their reliability in detecting renal scarring compared to established imaging standards. Research studies were carefully identified and filtered from three databases: PubMed, ScienceDirect, and Cochrane. The review aimed to evaluate the accuracy and effectiveness of biomarkers in detecting renal scarring in children with vesicoureteral reflux (VUR).

## Study eligibility criteria

In October 2024, a comprehensive search strategy was developed and executed using relevant keywords and Boolean operators. The search terms included combinations such as: “children”, “pediatric”, “vesicoureteral reflux”, “VUR”, “biomarkers”, “blood biomarkers”, “urine biomarkers”, “renal scarring”, “DMSA scan”, “dimercaptosuccinic acid scan”, “traditional imaging”, “accuracy”, “sensitivity”, “specificity”. Boolean operators (AND, OR) were employed to optimize the inclusion of studies aligned with the PICO framework (Population, Intervention, Comparison, Outcome). The search was limited to English-language studies, but no other language restrictions were applied. Inclusion criteria encompassed studies focusing on children diagnosed with vesicoureteral reflux, the use of blood or urine biomarkers for detecting renal scarring, comparisons with traditional imaging methods (e.g., DMSA scans), and studies reporting quantitative outcomes such as sensitivity, specificity, or overall accuracy. Exclusion criteria included studies involving adult populations, literature reviews, conference abstracts, case reports, and articles lacking quantitative data. Additionally, reference lists of selected articles were manually screened to ensure comprehensive coverage of relevant studies.

## Data extraction

Two independent reviewers extracted the following data from eligible studies to minimize bias: author and publication year, study design and sample size, population characteristics (e.g., age, VUR diagnosis), types of biomarkers assessed, comparators used (e.g., DMSA scans), and outcome measures- sensitivity, specificity, and accuracy in detecting renal scarring. Discrepancies between reviewers were resolved through consensus.

## Quality assessment and data synthesis

Quality assessment of the included study was conducted using the QUADAS-2 framework for diagnostic accuracy studies (7). Focusing on four key domains: patient selection, index test, reference standard, and flow and timing. In the patient selection domain, the tool assessed whether studies used consecutive or random sampling and applied appropriate exclusions. For the index test, it evaluated whether biomarker tests were interpreted without knowledge of the reference standard and whether diagnostic thresholds were pre-specified. The reference standard domain examined whether the gold standard test (typically DMSA) accurately classified the presence of renal scarring. The flow and timing domain reviewed whether all participants were included in the final analysis and whether the interval between index and reference tests was appropriate. The findings were summarized in tables and described narratively. Meta-analysis was not conducted due to variability in methodologies and outcomes.

## Results

### Study selection

**Figure 1:**
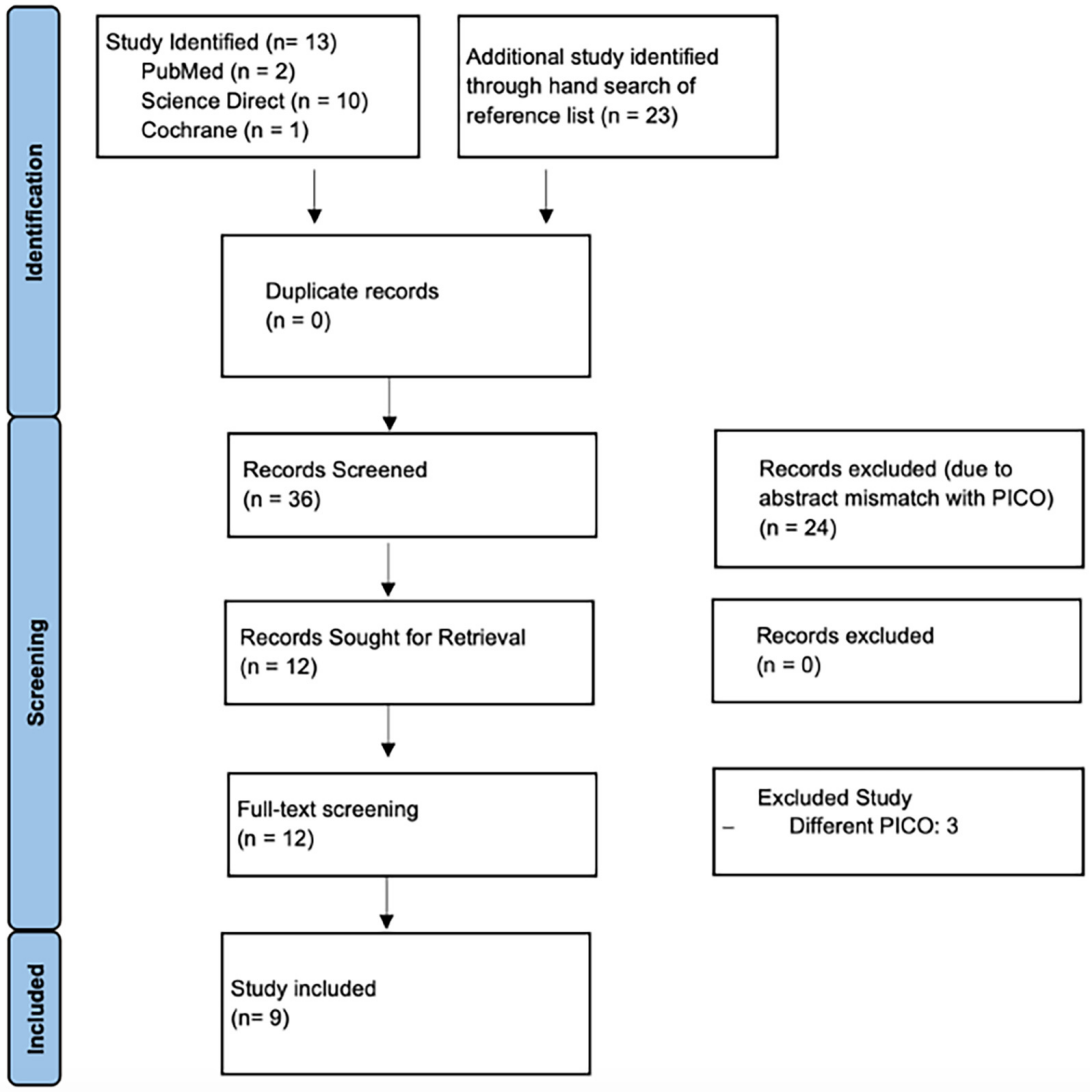
Prisma flowchart diagram for systematic review.

A comprehensive literature search was conducted across three databases, resulting in the identification of 13 studies. Additionally, a hand search of reference lists yielded 23 more studies, bringing the total to 36 records for screening. No duplicates were found. After the initial screening of titles and abstracts, 24 studies were excluded due to mismatches with the PICO criteria. The remaining 12 records were sought for retrieval, and all were successfully retrieved for full-text screening. During this stage, three studies were excluded for having different PICO criteria, leading to the final inclusion of 9 studies in the qualitative synthesis. The PRISMA flowchart (Figure 1) visually represents this selection process.

### Study characteristics and risk of bias

The nine included studies primarily focused on evaluating the diagnostic potential of urinary biomarkers for identifying VUR and renal scarring in pediatric populations (Table 1). These studies varied in design, with Nickavar et al (8), and Colceriu et al (9), adopting case-control methodologies, while Parmaksiz, et al. (10) and Mahyar et al (11), conducted observational cohort studies. Another studies by Bressan, et al. (12), Ohta, et al. (13), Gokce, et al. (14) and Gunasekara, et al. (15) and Islekel, et al. (16) conducting all conducted prospective cohort studies. Sample sizes ranged from 28–2,100 children, including both patients diagnosed with VUR and healthy control groups. Three studies examined urinary neutrophil gelatinase-associated lipocalin (NGAL), either in its absolute value or its relative ratio to the urinary creatinine level; whereas one study each investigated cathelicidin (LL-37), C-reactive protein (CRP), procalcitonin (PCT), interleukin 1 receptor-like 1 (ST2), interleukin-6 (IL-6), interleukin-8 (IL-8), cystatin, and urinary creatinine. Seven studies used voiding cystourethrography (VCUG) as the reference standard for diagnosing VUR, and five used DMSA to diagnose renal scarring. All studies reported outcomes relevant to diagnostic sensitivity, specificity, and overall accuracy.

**Table 1:** Study characteristics and outcomes.

Author(s); Year	Study Location	Study Design	Sample Size	Index Biomarker(s)	Reference Standard	Outcome^a^	Sensitivity (%)	Specificity (%)	AUC (95% CI)
Nickavar et al., (8)	Iran	Case-Control	69 children	NGAL/Cr	VCUG and DRC	VUR	84	81	0.86 (0.76–0.96)
Colceriu et al., (9)	Romania	Case-Control	78 children	NGAL/Cr	VCUG	Severe VUR	NA	NA	0.72 (0.54–0.90)
				LL-37/Cr			NA	NA	0.71 (0.55–0.88)
Mahyar et al., (11)	South Korea	Observational	153 children	CRP	VCUG	VUR	61	75	NA
Parmaksiz et al., (10)	Turkey	Prospective Cohort	123 children	NGAL	VCUG and DMSA	Renal scarring in primary VUR	72	60	0.74 (0.66–0.82)
Bressan et al., (12)	Italy	Prospective Cohort	72 children	PCT	VCUG and DMSA	Renal scarring in UTI	78.6	63.8	0.74 (0.61–0.85)
Ohta, et al., (13)	Japan	Retrospective Cohort	28 children	ST2	DMSA	Renal scarring in UTI	92.9	64.3	0.79
Gokce, et al., (14)	Turkey	Prospective Cohort	114 children	IL-6/Cr	VCUG and DMSA	VUR	45	80	0.65
				IL-8/Cr		Renal scarring	85	44	0.67
Islekel, et al., (16)	Turkey	Prospective Cohort	28 children	Cystatin	VCUG and DMSA	Renal scarring	NA	NA	NA
Gunasekara, et al., (15)	Sri Lanka	Cross-sectional	2,100 children	Urinary creatinine	NA	NA	NA	NA	NA

^a^ In all studies, classification of the severity of VUR was determined according to the International Reflux Study Group classification (grade I-V) (17).

CRP, C-reactive protein; DMSA, 99mTc-dimercaptosuccinic acid scintigraphy; DRC, direct radionuclide cystography; LL-37, cathelicidin; IL-6, interleukin 6; IL-8, interleukin-8; NGAL, neutrophil gelatinase-associated lipocalin; NGAL/Cr, neutrophil gelatinase-associated lipocalin/creatinine ratio; PCT, procalcitonin; ST2, interleukin 1 receptor-like 1; UTI, urinary tract infection; VUR, vesicoureteral reflux.

**Table 2:** Risk of bias of included studies using QUADAS-2 tools.

Study	Patient Selection	Index Test	Reference Standard	Flow and Timing	Overall Risk of Bias
Nickavar et al., (8)	Low	Low	Low	Low	Low
Colceriu et al., (9)	Moderate (casecontrol design)	Low	Low	Low	Moderate
Mahyar et al., (11)	Low	Moderate (threshold not pre-specified)	Low	Low	Moderate
Parmaksiz, et al., (10)	Low	Low	Low	Unclear	Low
Bressan, et al., (12)	Low	Low	Moderate	Low	Low
Ohta, et al., (13)	Low	Low	Low	Low	Low
Gokce, et al., (14)	Low	Moderate	Low	Low	Moderate
Islekel, et al., (16)	Low	Low	Low	Low	Low
Gunasekara, et al., (15)	Low	Low	Moderate	Low	Moderate

Using the QUADAS-2 tool to assess the risk of bias, the quality of the included studies varied (Table 2). Nickavar et al. and Colceriu et al. exhibited moderate risk of bias due to incomplete reporting of follow-up procedures and unclear blinding during biomarker analysis, which could introduce measurement bias (8, 9) Conversely, Mahyar et al. demonstrated low risks of bias, characterized by robust methodologies such as blinded biomarker evaluations and clearly defined inclusion and exclusion criteria (11). Parmaksiz et al., in particular, used well-established biomarkers like NGAL, KIM-1, and L-FABP to assess their potential in diagnosing reflux nephropathy, a severe complication of VUR. These studies minimized confounding by employing strict protocols for patient selection and objective measures such as quantitative biomarker analysis and imaging outcomes (10). While two studies faced limitations in their methodological rigor, all studies provided valuable insights into the diagnostic utility of urinary biomarkers, reducing detection bias by integrating objective measures with standardized analyses.

### Study outcomes

The outcomes of the included studies highlight the promising diagnostic utility of urinary biomarkers for the non-invasive detection of the association between vesicoureteral reflux (VUR) and renal scarring in children. Among these biomarkers, urinary NGAL/uCr consistently emerges as the most effective, demonstrating high sensitivity and specificity across multiple studies. Nickavar et al. (8) reported a sensitivity of 84%, specificity of 81%, and an area under the curve (AUC) of 0.86 for NGAL/uCr, supporting its accuracy in detecting VUR and renal damage. Parmaksiz et al. (10) also observed significantly elevated urinary NGAL levels in children with renal scarring, further supporting its diagnostic potential in identifying kidney damage in VUR patients. These findings position NGAL as one of the most reliable biomarkers for non-invasive diagnosis, with strong performance metrics comparable to traditional imaging modalities.

Other biomarkers, including CRP and LL-37, demonstrated moderate diagnostic performance. Mahyar et al. (11), found CRP levels (≥20 mg/dl) to have a sensitivity of 61% and specificity of 75% for detecting Renal Scarring in VUR, suggesting that while CRP may have limited utility as a standalone marker, it could enhance diagnostic accuracy when combined with imaging techniques. Colceriu et al. (9), reported NGAL/creatinine and LL-37/creatinine ratios as moderately effective, with AUC values of 0.72 and 0.71, respectively, particularly for identifying severe renal scarring in VUR. However, their diagnostic precision was lower compared to NGAL/uCr, indicating that these markers may serve complementary roles in conjunction with other diagnostic methods. Parmaksiz et al. (10) also found that urinary NGAL levels were significantly higher in patients with renal parenchymal scarring compared in those without, further confirming NGAL's superiority in detecting VUR-related kidney damage. Across the studies, urinary NGAL consistently showed superior diagnostic performance, while CRP, LL-37, and CXCL8/IL-8 served as additional tools to refine detection strategies. These results highlight the potential of urinary biomarkers to either replace or augment traditional imaging methods, thereby reducing reliance on invasive procedures like DMSA and VCUG while maintaining diagnostic accuracy.

Ohta et al. (13) indicated that serum soluble ST2 had a sensitivity of 92.9%, specificity of 64.3%, and an area under the curve (AUC) of 0.79 for forecasting renal scarring in children with upper urinary tract infections (UTI). These findings underscore soluble ST2 as a robust diagnostic biomarker for the early identification of renal scarring. Bressan et al. also identified PCT values exceeding 1 ng/ml as indicative of renal scarring, demonstrating a sensitivity of 78.6%, specificity of 63.8%, and an AUC of 0.74. This suggests that PCT can serve as a reliable biomarker for identifying children at risk of long-term renal impairment following acute pyelonephritis.

Gunasekara et al. (15) assessed urinary biomarkers, including KIM-1 and NGAL, in a large cohort study. Their findings revealed that the absolute levels of KIM-1 and NGAL reliably indicated tubular dysfunction, with little benefit from creatinine correction. KIM-1 and NGAL serve as important biomarkers for the early detection of kidney damage in juvenile populations. Gokce et al. (14) examined cytokines IL-6 and IL-8, revealing that IL-6/creatinine ratios were significantly higher in children with VUR compared to those without (median 5.72 vs. 3.73; *p* < 0.05). Additionally, the IL-8/creatinine ratios were elevated in children with renal scarring compared to those without (median 43.12 vs. 16.36; *p* < 0.05), and IL-8 levels correlated with the severity of scarring. These findings suggest that IL-6 and IL-8 may serve as non-invasive biomarkers for identifying inflammatory processes associated with VUR and renal scarring. Conversely, Islekel et al. (16) discovered that blood and urine cystatin C levels did not significantly differentiate between patients with renal scarring and those without. Cystatin C/creatinine ratios showed a correlation with indicators of tubular dysfunction, including urine NAG and microalbumin (*p* < 0.05). This suggests that cystatin C may indicate tubular damage rather than directly indicating renal scarring.

## Discussion

This systematic review evaluated the potential of urinary biomarkers as diagnostic tools for detecting renal scarring secondary to vesicoureteral reflux (VUR) in children. Biomarker itself can be defined as a characteristic that is objectively measured and evaluated as an indicator of normal biological process or pathogenic processes. It can be used as screening, diagnosis or even monitoring disease activity (18). The findings highlight those biomarkers such as NGAL, LL-37, IL-6, and CRP offer promising non-invasive alternatives to traditional imaging techniques. Among these, NGAL consistently showed the highest diagnostic performance. Studies by Nickavar et al. (8) demonstrated that NGAL had sensitivities ranging from 84%–90% and specificities from 81%–85%, with an area under the curve (AUC) of 0.86–0.88, confirming its reliability in detecting renal scarring and severe VUR (5, 6). NGAL's utility lies in its rapid secretion during renal injury, reflecting acute stress and inflammation in the kidneys. These findings align with broader research, such as one study by Jeong et al. which also highlights NGAL's correlation with tubular damage in children biomarkers demonstrated varying diagnostic capabilities (7). LL-37, a peptide involved in immune defence during urinary tract infections, showed moderate accuracy. As reported by Colceriu et al., the LL-37/creatinine ratio achieved an AUC of 0.71, indicating some potential for identifying severe VUR but limited utility as a standalone marker supported by its role in modulating inflammation and promoting tissue repair (9). Meanwhile, CRP, a common marker of systemic inflammation, demonstrated moderate sensitivity (61%) and specificity (75%), as shown by Mahyar et al. (11).

While CRP is less specific to VUR, its use as a complementary tool alongside imaging may enhance diagnostic accuracy. Multiple biomarkers, as suggested by Colceriu et al., may offer a more comprehensive diagnostic approach, as multi-marker panels can compensate for the individual limitations of each biomarker. Despite the promising potential of urinary biomarkers, notable challenges exist. The sensitivity and specificity of these biomarkers vary significantly due to differences in patient populations, assay thresholds, and reference standards. While NGAL consistently shows superior diagnostic performance, its utility in routine practice depends on the standardization of testing protocols and addressing pre- analytical variables like sample handling. Furthermore, although NGAL/uCr ratios demonstrate robust accuracy across studies, the absence of longitudinal follow-up data limits conclusions about their use in monitoring disease progression or treatment response. Similarly, markers like LL-37 and CRP require further validation in larger and more diverse cohorts to confirm their clinical utility.

Procalcitonin (PCT) was also identified as a reliable biomarker for predicting renal scarring, with the study by Bressan et al. (12) study indicating its utility in acute settings such as pediatric pyelonephritis. Additional reviews, such as those by Mattoo et al. (19), further corroborate PCT's diagnostic potential, particularly in differentiating acute pyelonephritis (APN) from lower UTIs.

Urinary cytokines IL-6 and IL-8 demonstrated potential as non-invasive markers. These findings align with the study by Mattoo et al. (19), which reported AUC values of 0.89 for IL-6 and 0.95 for IL-8 in the detection of febrile UTIs. Soluble ST2, a serum biomarker, exhibited high sensitivity (92.9%) and moderate specificity (64.3%) for predicting renal scarring in pediatric patients with upper UTIs, as reported by Ohta et al. The ability of this marker to identify children at risk for long-term renal damage offers a promising diagnostic avenue. In contrast, cystatin C, evaluated by Islekel et al. (16), showed limited utility in distinguishing between scarred and non-scarred kidneys but correlated with markers of tubular dysfunction, such as microalbumin and NAG (*p* < 0.05). This finding suggests a potential role in identifying early tubular injury rather than directly observing scarring.

The study by Naik et al. indicated that a single NGAL measurement is insufficient, and continuous monitoring of NGAL levels in children with recurrent UTIs may serve as a valuable indicator for assessing the progression of renal scarring (18). The study by Forster and Devarajan (5, 20) indicated that the disparity in NGAL values between patients with scarring and those without is statistically significant. There are two variants of NGAL with distinct applications based on their upregulation mechanisms: pNGAL, which functions as a marker for systemic inflammatory diseases, and uNGAL, which is exclusive to renal epithelial injuries. A study by Yilmaz et al. indicates that the CRP value is markedly elevated upon admission when renal impairment is identified with DMSA following pyelonephritis. The study revealed a greater incidence of renal scarring in patients exhibiting elevated CRP values, with a statistically significant difference identified (21).

Although NGAL and PCT exhibit consistently higher diagnostic efficacy, markers such as IL- 6, IL-8, and soluble ST2 serve as complementary tools for specific situations, including the differentiation of inflammation-driven disorders like VUR. Nonetheless, challenges persist, including inconsistencies in diagnostic thresholds, test methodologies, and patient demographics. The standardization of biomarker-based diagnoses is complicated by these factors, as Mattoo et al. emphasized the dual utility of these biomarkers in providing immediate diagnostic insights and guiding therapeutic decisions (19). PCT, with a threshold of ≥0.5 ng/ml, demonstrates sensitivity and specificity ranging from 70%–86% and 76%–91%, respectively, in differentiating acute pyelonephritis (APN) from lower UTI. PCT concentrations correlate with the severity of kidney lesions, offering prognostic value for subsequent renal scarring when integrated with imaging findings, such as DMSA scans.

## Study limitations

This systematic review synthesized data from nine studies; however, the methodological variability and reliance on case-control designs in two studies limited the generalizability of the findings. Observational and case-control studies are prone to selection bias, which restricts the ability to establish causal relationships. Small sample sizes and the absence of robust longitudinal follow-up further constrained the evaluation of biomarkers' utility over time. Differences in biomarker assay methods and thresholds introduced heterogeneity, complicating direct comparisons across studies and reducing the feasibility of meta-analysis. Finally, while NGAL demonstrated promising diagnostic accuracy, its role in predicting disease progression or monitoring treatment responses remains underexplored.

## Conclusions

This systematic review underscores the diagnostic potential of urinary biomarkers for detecting renal scarring secondary to VUR in pediatric populations. NGAL, with its high sensitivity, specificity, and diagnostic accuracy, emerged as the most promising biomarker, capable of complementing imaging techniques such as DMSA. LL-37 and CRP showed moderate utility as adjunct biomarkers, while multi-biomarker approaches demonstrated potential to enhance diagnostic precision. Despite these advances, the findings emphasize the need for high-quality randomized controlled trials with larger sample sizes, standardized biomarker thresholds, and robust follow-up protocols. Future studies should explore the long-term utility of biomarkers in monitoring disease progression and assessing therapeutic responses, paving the way for a more precise and non-invasive diagnostic framework for renal scarring and VUR.

## Data Availability

The original contributions presented in the study are included in the article/Supplementary Material, further inquiries can be directed to the corresponding authors.
